# Nutritional Status and Associated Factors among Adult Psychiatric Patients in Dessie Referral Hospital, Northeast Ethiopia

**DOI:** 10.1155/2020/5087573

**Published:** 2020-04-03

**Authors:** Tesfaye Assefa, Omer Seid, Fentaw Tadese, Tsegaye Gebremedhin

**Affiliations:** ^1^Department of Public Health, Wollo University, Dessie, Ethiopia; ^2^Department of Nutrition and Dietetics, School of Public Health, College of Medicine and Health Sciences, Bahir Dar University, Bahir Dar, Ethiopia; ^3^Department of Health Systems and Policy, Institute of Public Health, College of Medicine and Health Sciences, University of Gondar, Gondar, Ethiopia

## Abstract

**Background:**

Psychiatric illnesses involve alterations in the brain or nervous system function and result in altered perception, responses to the environment, and daily functioning, which ultimately diminish the capacity to cope with the regular demands of life, including nutritional intake. Little is known about nutritional status in psychiatric patients in our setup, particularly in northeast Ethiopia. Thus, this study is aimed at assessing the nutritional status and associated factors among adult psychiatric patients in Dessie referral hospital, northeast Ethiopia.

**Methods:**

A facility-based cross-sectional study was employed among 530 psychiatric patients using an interviewer-administered structured questionnaire, and anthropometric measurements were taken from April 10 to June 20, 2018. Multinomial logistic regression analysis was used to identify the associated factors with the nutritional status of adult psychiatric patients.

**Results:**

The study revealed that the proportion of undernutrition and overnutrition was 20.0% (95% CI: 16.80-23.60) and 23.4% (95% CI: 19.80-27.00), respectively. Being male (AOR: 2.39, 95% CI: 1.28-4.47), private employed (AOR: 0.08, 95% CI: 0.02-0.31), and not consuming alcohol (AOR: 0.20, 95% CI: 0.56-0.74) were factors associated with undernutrition. Whereas not involved in physical activity (AOR: 2.98, 95% CI: 1.37-6.49), being newly diagnosed patient (AOR: 1.86, 95% CI: 1.01-3.42), and not chewing *Khat* (AOR: 3.92, 95% CI: 1.63-9.42) were factors associated with overnutrition of adult psychiatric patients.

**Conclusion:**

The proportion of both undernutrition and overnutrition was above the national average. Notably, nutrition significantly affects the production and use of neurotransmitters, and this may result in significant effects on physical, mental, or emotional processes. This undoubtedly affects the stabilization processes of neuropsychiatric patients. Therefore, healthcare managers, supervisors, and policymakers identify those vulnerable groups early and thereby design effective nutritional strategies to intervene in malnutrition among adult patients with a psychiatric disorder.

## 1. Introduction

Diet and nutrition have got tremendous impact in preventing disease and reducing the morbidity and mortality of many diseases. The nutritional status of individuals is an essential factor affecting mental health and the development of psychiatric disorders [1].

According to the World Health Organization (WHO), International Classification of Disease (10^th^ edition) known as ICD-10; mental, behavioral, and neurodevelopmental disorders are the psychological development disorders. Psychiatric disorders are classified as psychoactive substance use, schizophrenia and delusional, affective (mood), neurotic stress-related and somatoform, behavioral syndromes associated with physiological disturbance, and disorders of adult personality and behavior [2].

Psychiatric illnesses involve alterations in the brain or nervous system function and result in altered perception and responses to the environment [3]. The illnesses disrupt a person's thinking, feeling, mood, ability to relate to others, and daily functioning, which ultimately diminish the capacity to cope with the regular demands of life, including nutritional intake [4].

Several psychiatric illnesses, including schizophrenia, mood disorders, eating disorders, and substance abuse, adversely affect food intake and nutritional status. The drugs used to treat those disorders similarly have effects on appetite and gastrointestinal function and interact with food and nutrients [5]. Psychiatric illness seldom increases nutrient requirements, but there are many reasons for high incidence of poor nutrition among mentally ill patients [6], including neglected diet during a period of emotional stress, depression, disinterest, forgetfulness, confusion, and anxiety, all of which can cause a decrease in food intake and lead to nutritional deficiency.

Patients with impaired mental health are strongly vulnerable to the risks of having involuntary weight gain, weight loss, or deficiency of essential nutrients [5]. Poor nutrition is associated with a greater chance of the occurrence of many diseases and adverse health outcomes, such as sarcopenia, frailty syndrome, and disability [7]. The current practice of psychiatry is undermined by the insufficient biological understanding of mental health disorders, underrecognition, and stigmatization, lack of effective therapeutic interventions, and lack of access to care delivery, including nutritional rehabilitation [8].

The prevalence of undernutrition among patients with major depressive disorders (MDD) in northwest Ethiopia is high. Residences, multiple medications, taking prescribed diet, and current use of alcohol were factors associated with undernutrition among depressive patients [9]. Similarly, these factors may influence the nutritional status of those psychiatric patients in the study area, and the prevalence or magnitude of the problem is not determined in this locality. Moreover, psychiatric illness affects the functioning of an individual in society and, overall, determines the state of health and the quality of life [10].

Nutritional status among psychiatric patients is not given attention even in the sustainable development goals and health sector transformation plan. Prevention, as well as early detection and treatment of under and overnutrition among psychiatric patients, is not well addressed. However, despite the high burden of mental disorder and nutritional problems, little research has been done regarding the prevalence and associated factors of nutritional status among adult psychiatric patients in Ethiopia [9].

Therefore, assessing nutritional status and the associated factors among psychiatric patients is crucial to provide a holistic approach intervention, include nutrition-related health indicators, dietary intake, and energy expenditure [11]. Therefore, this study has assessed the proportion of nutritional status and associated factors among adult psychiatric patients attending outpatient departments (OPDs) of the Dessie referral hospital psychiatric clinic in 2018.

## 2. Methods and Materials

### 2.1. Study Design and Setting

The facility-based cross-sectional study design was conducted at Dessie referral hospital, which is located in Dessie town, Amhara National Regional State, from April 10 to June 20, 2018.

Dessie town is the capital of the south Wollo zone in the Amhara region, located 401 kilometers away from Addis Ababa to Northeast Ethiopia. Based on the 2007 national census conducted by the Central Statistical Agency (CSA) of Ethiopia, Dessie town has a total population of 205,558. Of those, 46.1% were males. There are two public hospitals, eight health centers, and six health posts. Moreover, there are also two nongovernmental organization (NGO) medium clinics, three general hospitals, two specialty centers, seven higher clinics, and 13 lower clinics which are privately owned.

Dessie referral hospital has three psychiatric OPDs. Four Bachelor of Sciences (BSc) in psychiatric nurses and two clinical psychiatrists were providing the service. A total of 7,545 psychiatric cases were treated in 2017, according to the hospital's health management information system (HMIS) report.

### 2.2. Population and Sampling Procedures

The source populations were all adult psychiatry patients who had been attending in Dessie referral hospital psychiatric OPDs. Whereas all selected adult psychiatric patients attended in Dessie referral hospital psychiatric OPD during the data collection period, and patients who participated were the study population.

All adult psychiatric patients attending Dessie referral hospital psychiatric OPDs were included in this study. Whereas, adult psychiatric patients with physical deformity (kyphosis or lordosis), edema, pregnant, lactating women (less than six months), and psychiatric emergency were excluded from the study.

The sample size was calculated by using both single and double population proportion formula. The sample size for the first objective (prevalence) was calculated by using single population proportion formula; with an assumption of proportion (*P*) of MDD patients having overnutrition was 50% [9], 5% margin of error (*d*), 95% confidence level, and adding 10% nonresponse rate. *N* = (*Z*_*α*/2_)^2∗^*P* (1 − *P*)/(*d*)^2^ = (1.96)^2^(0.5) (0.5)/(0.05)^2^ = 384. After adding 10% nonresponse rate, it gave 423.

The sample size for the second objective (factors) was calculated using the double population proportion formula by taking strongly significant variables in previous studies. The proportion of MDD patients residing urban area and having undernutrition was 40.3%, using alcohol and having undernourished was 34%, and taking multiple medications and having undernourished was 32.3% [9], with the power of 80%, 95% confidence level, and 10% nonresponse rate. Accordingly, the calculated sample size was 405, 159, and 559, respectively, for each significant factor, and the sample size for the third factor was larger than the others. Therefore, for maintaining sample size adequacy, it was better to use the largest sample size, which was 559. Finally, the study participants were selected using a consecutively sampling technique, and trained data collectors performed the interview and anthropometric measurements.

### 2.3. Variables and Measurement

Nutritional status was the dependent variable of the study, whereas sociodemographic and economic factors (age, sex, educational status, marital status, religion, ethnicity, monthly income, occupational status, and living condition (with family, alone)), health-related factors (types of visit, main diagnosis, types of drugs taken, treatment duration, acute illness, loss of appetite, and chronic illness), and behavioral factors (use of alcohol, substance use, smoking, and physical activity) were the independent variables of the study.

Body mass index (BMI) weight of the individual in kilogram is divided by the square of the height in meter and used to determine the nutritional status of psychiatric patients and classified as follows: undernutrition (BMI < 18.49 kg/m^2^), normal weight (BMI = 18.5 − 24.99 kg/m^2^), and overnutrition (BMI ≥ 25.0 kg/m^2^) [12].

Dietary diversity was measured using the sum of food group/items consumed by the study participant [13, 14] and based on this, it was classified as follows:

Low: those who consumed three and less from the nine food groups/items

Medium: those who consumed four to six items from the nine food groups/items

High: those who consumed seven to nine items from the nine food groups/items

Psychiatric emergency is a situation in which a psychiatric patient cannot be refrained from acting in a manner that is dangerous to others; as a result, taking weight and height is impossible.

A new visit is defined as an adult patient who had a psychiatric problem and found in Dessie referral hospital psychiatric OPD for the first time during the data collection period.

In repeat visit, there is an adult psychiatric patient who had more than one follow-up in Dessie referral hospital psychiatric OPD at the time of data collection.

In household food security, if all answers for the 9 household food access score (HHFAS) items are no food secured, otherwise food insecure [15].

In Khat chewer, there is a patient who chewed Khat within a month [16].

Sleeping pattern was measured when a patient reports their sleeping pattern as increased or decreased compared to the previous normal pattern. So, it was classified as normal, increased, or decreased.

### 2.4. Data Collection Tools and Procedures

An interviewer-administered structured questionnaire adapted from different studies [5, 8, 9, 15, 17–22] was used to collect data among adult psychiatric patients attending OPDs of a psychiatric clinic.

Standard food and nutrition technical assistant (FANTA) tools were used for dietary diversity questionnaires based on 24-hour recall from subjects or caregivers. The questionnaires have a score of 0-9 (0 − 3 = low dietary diversity score (DDS), 4 − 6 = medium DDS, and 7 − 9 = high DDS).

Food security was assessed using the household food insecurity access scale (HFIAS), a tool validated in other developing countries. The HFIAS has nine questions asking the household's last month's experience about three domains of food insecurity: feeling the uncertainty of food supply, insufficient quality of food, and insufficient food intake and its physical consequences. Study participants were categorized into two levels of food-security status (food-secure and food-insecure).

Height and weight measurements were done in accordance with the WHO study of global aging and adult health (SGAE) [23]. Alcohol consumption, tobacco, and other substance use were assessed and categorized based on the WHO stepwise approach to chronic disease risk factor for surveillance [17]. Work-related and leisure/spare time physical activity was also measured by global physical activity questionnaire (GPAQ) and SGAE [17, 23, 24].

Bodyweight of study subject was measured using a SECA digital weight scale. The study subject's worn only light clothes and being without shoes during the measurement of body weight.

Besides, height was measured using a measuring board with a precision of 0.1 cm. The head of the study participant was in the Frankfurt position (a position where the line passing from the external ear hole to the lower eyelid of a person is parallel to the board).

The questionnaire was initially developed in English and translated into the local language (Amharic) and finally back to English in order to ensure its consistency. Six trained BSc nurses for data collectors and two BSc in public health supervisors were recruited. The training was given for one day on the basic techniques of data collection, and pretesting was conducted on 28 psychiatric patients at Boru Meda hospital before the actual data collection. Finally, all findings from the pretest were incorporated into the final questionnaire. During data collection, supervisors have checked the data daily for accuracy, consistency, and completeness.

### 2.5. Data Management and Analysis

Data were cleaned, coded, and entered to EpiData version 3.1 and exported to the Statistical Package for Social Sciences (SPSS) version 22 software for analysis. Frequencies and percentages of variables were presented using tables and graphs. Bivariable and multivariable logistic regression analysis was conducted, and those predictor variables with a *P* value less than 0.25 during bivariable analysis were entered to the final multinomial logistic regression analysis. Adjusted odds ratio (AOR) with 95% confidence interval (CI) and *P* value was used to identify the strength and associated factors with the outcome variables. Those predictor variables with a *P* value of less 0.05 in the final multinomial logistic regression analysis were used to declare the significant variables.

### 2.6. Ethical Considerations

Ethical approval was obtained from the Institutional Research Review Board of Wollo University after approving the procedure (*Ref. No. CMHS2909/13/10*), and permission letter was also obtained from Dessie referral hospital. The investigators explained to the participants the procedures, risks, and benefits of a study. Moreover, investigators are also ensured that participants understood this information so that they can decide voluntarily whether they want to participate. Finally, written consent was obtained from each study participants and their parents/guardians for those who had difficulty to communicate to ensure their voluntariness to participate in the study, and all participants had a right to withdraw at any time or to skip for a single question or segment of questions. Participant's privacy and confidentiality of the information were maintained through nonidentifiers of the name.

## 3. Results

### 3.1. Sociodemographic and Economic Characteristics of Study Participants

A total of 530 psychiatric patients have participated in the study, with a response rate of 94.8%. The mean age of the respondents was 27.9 ± 8.8 years, and the minimum and maximum years of the participants were 18 and 60, respectively. Two hundred sixty-six (50.2%) were male, and 278 (52.5%) were married. Religiously, 290 (54.7%) of patients were Muslim followers. More than three-fourths (82.8%) of the study participant's ethnicity were Amhara; similarly, 439 (82.8%) of them were living with family. Three hundred six (57.7%) of the participants were from a family size of five and above. The average monthly income of the household was 1858.40 Ethiopian birr ($66.3 US). Regarding educational status, 152 (28.7%) of patients were unable to read and write, and 139 (25.5%) were able to read and write. Among the study participants, 197 (37.2%) were farmers (Table 1).

### 3.2. Feeding Habit and Dietary Diversity

Around three-fourths (74.2%) of the respondents had eaten their breakfast in the morning. Of these, 215 (54.7%) used to take their breakfast always except fasting day. Regarding the mode of feeding, 380 (71.7%) of psychiatric patients feed themselves without any problem. *Teff* is the main staple food in 336 (63.4%) study participants and almost half; 247 (46.6%) of the study respondents' source of main staple food is through purchasing. The majority of the respondents, 381 (71.9%), had no habit of taking a snack, and 375 (70.8%) used to take meals three times per day other than a snack (Table 2).

The dietary diversity was measured using the sum of nine food groups/items consumed by the participants. Accordingly, 89 (16.8%), 290 (54.7%), and 151 (28.5%) of participants were with high, medium, and low dietary diversity scores, respectively. For 223 (42.0%) patients', food was secured.

### 3.3. Physical Activity and Sleeping Pattern

One hundred fifty-eight (29.8%) of participants performed vigorously (running, football, digging, carrying, and lifting) physical activity with an average of 2.89 days per week, and the mean time spent were 51.65 minutes. Moreover, 196 (37.0%) of participants perform moderate (brisk walking, cycling, swimming, volleyball) physical activity averagely 2.42 days per week with a mean time of 30.47 minutes spend per each session. On average, patients spend 104.3 minutes inactively or laying down on a typical day. Of the total participants, 190 (35.9%) had normal sleeping pattern; and for the 271 (51.1%) and 69 (13.0%), their sleeping pattern was increased and decreased, respectively.

### 3.4. Health-Related Factors

More than half of the psychiatric patients 274 (51.7%) were on follow-up/repeat visits, and the rest were newly visited. Of those, one hundred seventeen (22.0%) patients were diagnosed with schizophrenia, followed by MDD (21.7%) and a bipolar mood disorder (17.2%) (Table 3).

Seventy-three (13.8%) of the study participant had gotten nutrition-related counseling or intervention along with psychiatric treatment. The majority (91.6%) of the study participants were taking less than four kinds of prescribed drugs per day for their treatments from those who were on follow-up/repeat visit, and the rest take four and above kinds of prescribed drugs per day. Only 228 (43.0%) of them have been declining in food intake over the past three months. Of those, 178 (91.7%) were due to loss of appetite, and the rest were due to lack of food.

### 3.5. Substance and Alcohol Use Behavioral Factors

The majority, 345 (65.1%), of the study participants use substance and alcoholic beverages. Of these, 140 (26.4%) chew *Khat*, 88 (16.6%) smoke Cigarettes, 42 (7.9%) drink Beer, and 29 (5.5%) take *Araki* (Table 4).

### 3.6. Nutritional Status of Adult Psychiatric Patients

The average bodyweight of the study participant was 59.3 kg with a standard deviation (SD) of 11.2 kg. Moreover, the mean height was 164.6 cm (SD = 8.6 cm). Regarding their body mass index, the majority of the participants 300 (56.4%) were in the normal range, 106 (20.0%) were having undernutrition, and 124 (23.4%) were having overnutrition.

### 3.7. Factors Associated with Nutritional Status of Psychiatric Patients

In bivariable logistic regression analysis; sex, educational status, eating breakfast, nutritional counseling, acute illness, chronic illness, alcohol consumption, a habit of snack, physical activity, type of visit, sleeping pattern, *Khat* chewing, age of participants, and occupational status were found to be a candidate for multivariable logistics regression.

In the final model, factors significantly associated with undernutrition were sex, occupational status, and alcohol consumption. Accordingly, the odds of having undernutrition were 2.4 times more in males than females (AOR: 2.39, 95% CI: 1.28-4.47). The odds of being undernourished were 92.1% less in private employees than government employees (AOR: 0.08, 95% CI: 0.02-0.31), *P* value<0.001). Patients who do not take alcohol were 79.5% less likely to be undernourished (AOR: 0.205, 95% CI: 0.56-0.748) as compared to those who take alcohol.

However, factors significantly associated with overnutrition in the final model were the type of visit, *Khat* chewing, and not involving in moderate physical activity. Accordingly, the odds of having overnutrition were 1.86 times more in newly diagnosed patients than patients who were on follow-up (AOR: 1.86, 95% CI: 1.01-3.42). The probability of being overnourished was 2.99 times more among patients who were not involved in moderate physical activity than those who were doing moderate physical activity (AOR: 2.99, 95% CI: 1.37-6.49). Patients who do not chew *Khat* were 3.92 times more overnourished compared to those who chew *Khat* (AOR = 3.92, 95% CI: 1.63-9.42) (Table 5).

## 4. Discussion

The study finding showed that 20.0, 23.4, and 56.4% of adult psychiatric patients who attended the outpatient department of Dessie referral hospital were undernutrition, overnourished, and well-nourished, respectively.

The proportion of undernutrition is lower than a study conducted in northwest Ethiopia on prevalence and factors associated with undernutrition among adults with MDD shows that 31.4% were undernourished [9]. This lower result might be due to the difference in study participants, which consists only of MDD patients, but the current study includes all psychiatric patients.

However, the proportion of undernutrition in the study area is much higher than studies done in Japan on the prevalence of underweight and overweight/obesity, and laboratory data for nutritional status in Japanese outpatients and inpatient with schizophrenia (4.3%) [18]. This discrepancy might be due to variations in socioeconomic status, dietary habits, and health care access. Besides, this study was done among all psychiatric patients, whereas that of Japanese study was done only on schizophrenic patients.

From those psychiatric patients having undernutrition, 67 (63.2%) were males. This finding is consistent with a study conducted in Malawi on anthropometric characteristics, and the burden of altered nutritional status among neuropsychiatric patients at Zomba mental hospital had shown that male patients were significantly taller and heavier than females, but had a significantly lower mean BMI than female patients [20, 25].

Among patients having undernutrition, majority 45 (42.4%) were diagnosed with schizophrenia, followed by 19 (17.9%) anxiety disorder, and 17 (16.0%) MDD. This finding is consistent with a cross-sectional study conducted at Dhaka, Bangladesh, on the nutritional status of psychiatric patients attending OPD at a selected tertiary hospital, which showed that 42.0% were diagnosed with schizophrenia [21]. This similarity might be due to the study setup and population, which consists of all psychiatric patients.

The multinomial logistic regression analysis result revealed that the likelihood of being undernourished was found to be higher among male psychiatric patients than females, and privately employed psychiatric patients were 92.1% less likely to be undernourished than the government employed. Those patients who do not consume alcohol were 80.0% less likely to be undernourished when compared to psychiatric patients who consume alcohol.

Our study finding shows that male sex, alcohol consumption, and being government employees were significantly associated with undernutrition. A study conducted in northwest Ethiopia showed that current use of alcohol was also a factor associated with undernutrition among depressive patients [9]. This similarity might have resulted from the study area and nearly similar environmental factors between the study areas.

In the present study, male sex was significantly associated with having undernutrition; the odds of undernutrition among male psychiatric patients were nearly two times more likely compared to females. This finding is similar to another study done in northeastern Ethiopia on age-related factors influencing the occurrence of undernutrition among the general population [19]. This could be due to related cultural practices in the study area as well as in nationwide; males are more responsible members of the family in generating income and keeping the welfare of the family. As a result, males are more mobile and engaged in activities which could make them lose a greater amount of energy. Studies reported a regional and country-specific variation about the difference in the prevalence of undernutrition between males and females [26].

As this finding showed, adult psychiatric patients who did not consume alcohol in the last six months were 80.0% less likely to be undernourished than alcohol consumers. This finding is in line with a conducted study in northwest Ethiopia. This is because alcohol use interferes with the absorption and storage of micronutrients. Besides, alcohol irritates the gastrointestinal system; it increases acid secretion by the stomach, which can injure the lining of the small intestine and interferes with the ability to absorb vital nutrients. Furthermore, alcohol is a toxin that has to be deactivated by the liver, and during the detoxification process, the body uses thiamin, zinc, and other important nutrients. Additionally, it can deplete the investments of healthy metabolism and also cause low mood, irritability, and/or aggressive behavior as well as more serious and long-term mental health problems, which further predispose to undernutrition [9]. It is also in line with a study conducted in Malawi on anthropometric characteristics, and the burden of altered nutritional status among neuropsychiatric patients at Zomba mental Hospital had shown that alcohol use are known risk factors for undernutrition [25].

This finding also revealed that new psychiatric patients were twice more likely to be overnourished compared to those who had a follow-up. This could be due to patients who had to follow-up may get nutrition-related counseling or intervention or weight loss treatment. This finding is in line with a cross-sectional study conducted in Egypt at Al-Hussein University Hospital that had shown that the prevalence of obesity was high in new outpatients with mental disorders and was 47.2% in all patients [27]. It is also supported by the study findings from Burdur State Hospital, Turkey, and Iran that patients with abnormal nutritional status were found to have significant longer disease history [28, 29].

The odds of overnutrition were found to be three times higher among patients who did not involve in physical activity than those who were involved in physical activity. This might result from the inability to lose excess energy and excess body weight. The finding is in line with a study conducted in the US among adult population, which showed that obesity was associated significant with major depression [30]. The odds of having overnutrition among those psychiatric patients who did not chew Khat in the past one month were four times more than those Khat chewers may be due to the effect of Khat like; dental problem, laceration of the oral mucosa, gastritis, constipation, and weight loss. Also, the active ingredients of Khat (cathinone and cathine) have an appetite inhibiting effect on the hypothalamus [31]. This could be related to concurrent consumption of sugary drinks and sweets to counteract the bitter taste of *Khat*, coupled with reduced appetite, and delaying the effect of intestinal absorption of *Khat* users.

### 4.1. Strength and Limitations of the Study

Using adequate sample size and including all psychiatric patients with all types of psychiatric disorders in the study to see the nutritional status distribution among the type of psychiatric disorders were the strength of the study.

However, the study might be prone to social desirability bias as a result of using an interviewer-administered questionnaire. It did not also show the cause-effect relationship of nutritional status and psychiatric disorder because of its cross-sectional nature.

## 5. Conclusion and Policy Implications

The prevalence of under and overnutrition was found to be high in the study, which was above the national average. Occupational status, sex, and consuming alcohol were factors associated with undernutrition. Type of visit, performing physical activity, and chewing Khat were factors associated with overnutrition among psychiatric patients.

These psychiatric patients who are found severely malnourished, they are strongly connected with health care providers at that moment. Obese patients should be encouraged to participate in energy expenditure physical activity. After this, health workers consistently should provide nutritional counseling and weight management accompanying with their treatment for these psychiatric patients.

Notably, nutrition significantly affects the production and use of neurotransmitters, and this may result in major effects on physical, mental, or emotional processes. This undoubtedly affects the stabilization processes of neuropsychiatric patients. Findings from this study will help the policymakers to identify these target groups and thereby design effective nutritional strategies to intervene in malnutrition among adult patients with a psychiatric disorder.

## Figures and Tables

**Table 1:** Sociodemographic and economic characteristics of psychiatric patients in Dessie referral hospital, northeast Ethiopia, June 2018 (*n* = 530).

Variables	Description	Frequency (*n*)	Percent (%)
Sex	Male	266	50.2
	Female	264	49.8

Age in years	18-24	237	44.7
	25-35	195	36.8
	≥36	98	18.5

Marital status	Married	285	53.8
	Single	191	36.0
	Others^∗^	54	10.2

Religion	Muslim	290	54.7
	Orthodox	196	37.0
	Protestant	44	8.3

Residence	Urban	278	52.5
	Rural	252	47.5

Educational status	Unable to read and write	152	28.7
	Able to read and write	135	25.5
	Elementary school (grade 1-8)	128	24.1
	High school (grade 9-12)	71	13.4
	College and above	44	8.3

Occupational status	Farmer	197	37.2
	Private employee	88	16.6
	No job	74	14.0
	Merchant	51	9.6
	Gov't employee	42	7.9
	Daily laborer	41	7.7
	Others^∗∗^	37	7.0

Ethnicity	Amhara	439	82.8
	Afar	49	9.3
	Oromo	33	6.2
	Tigre	9	1.7

Living condition	With family	439	82.8
	Alone	91	17.2

Family size	≥5	306	57.7
	<5	224	42.3

Monthly income in Ethiopian birr (ETB)^∗∗∗^	< 1858.40	281	53.0
	≥1858.40	249	47.0

^∗^Others: divorced, widowed; ^∗∗^others: housewife, students; ^∗∗∗^the minimum monthly income in Ethiopia (for civil servants) was 650 ETB.

**Table 2:** Dietary habits of psychiatric patients in OPD of Dessie referral hospital, northeast Ethiopia, June 2018 (*n* = 530).

Variables	Descriptions	Frequency (**n**)	Percent (%)
Have you eaten your breakfast in the morning?	No	137	25.8
	Yes	393	74.2

How often do you eat breakfast?	Sometimes	4	11.7
	Always except fasting day	215	54.7
	Always	132	33.6

Mode of feeding	Unable to eat without assistance	33	6.2
	Self-fed with difficulty	117	22.1
	Self-fed without any problem	380	71.7

Main staple foods	Teff	336	63.4
	Sorghum	164	30.9
	Barley	30	5.7

Source of main staple foods	Own product	236	44.5
	Purchase	247	46.6
	Both own product and purchase	47	8.9

A habit of eating a snack	No	381	71.9
	Yes	149	28.1

Meals per day other than snack	Less than or equal to once	7	1.3
	Twice	81	15.3
	Three times	375	70.8
	Four times and above	67	12.6

**Table 3:** Distribution of patients by psychiatric diseases type in OPD of Dessie referral hospital, northeast Ethiopia, June 2018 (*n* = 530).

Types of psychiatric disease	Frequency (*n*)	Percentage (%)
Schizophrenia	117	22.0
Schizophrenia affective	51	9.7
Somatoform disorder (SD)	54	10.2
Anxiety disorder (AD)	65	12.2
Major depressive disorder (MDD)	115	21.7
Bipolar mood disorder (BMD)	91	17.2
Others^∗^	37	7.0

^∗^Epilepsy, seizure.

**Table 4:** Substance and alcoholic beverage ever used in the past six months by psychiatric patients in OPD of Dessie referral hospital, northeast Ethiopia, June 2018 (*n* = 345).

Substance and alcohol use behavior	Frequency (*n*)	Percentage (%)
Tobacco smoking	28	5.3
Cigarette smoking	88	16.6
*Khat*	140	26.4
Wine	18	3.4
Beer	42	7.9
*Araki*	29	5.5

**Table 5:** Factors associated with the nutritional status of adult psychiatric patients attending OPD of Dessie referral hospital, northeast Ethiopia, June 2018.

Variables	Nutritional status			Undernutrition		Overnutrition	
	Normal	Under	Over	COR (95% CI)	AOR (95% CI)	COR (95% CI)	AOR (95% CI)
	*n* (%)	*n* (%)	*n* (%)				
Sex							
Male	147 (49.0)	67 (63.2)	52 (41.9)	1.78 (1.13-2.81)^∗^	2.39 (1.27-4.47)^∗∗^	—	—
Female	153 (51.0)	39 (36.8)	72 (58.1)	1	1	1	1
Type of diagnosis							
New	145 (48.3)	38 (35.8)	73 (58.9)	—	—	1.53 (1.00-2.33)^∗^	1.86 (1.0-3.42)^∗^
Repeat	155 (51.7)	68 (64.2)	51 (41.1)	1	1	1	1
Physical activity							
No	184 (61.3)	58 (54.7)	92 (74.2)	—	—	1.81 (1.13-2.88)^∗^	2.99 (1.37-6.49)^∗∗^
Yes	116 (38.7)	48 (45.3)	32 (25.8)	1	1	1	1
Khat chewing							
No	112 (70.7)	72 (67.9)	106 (85.5)	—	—	2.44 (1.39-4.27)^∗∗^	3.92 (1.63-9.42)^∗∗^
Yes	88 (29.3)	34 (32.1)	18 (14.5)	1	1	1	1
Alcohol drinking							
No	294 (98.0)	92 (86.8)	115 (92.7)	0.13 (0.05-0.35)^∗∗^	0.20 (0.06-0.74)^∗^	—	—
Yes	6 (2.0)	14 (13.2)	9 (7.3)	1	1	1	1
Employment							
Private	56 (10.6)	6 (1.1)	26 (4.9)	0.18 (0.07-0.45)^∗∗^	0.08 (0.02-0.31)^∗∗^	—	—
Government	25 (4.7)	5 (0.9)	12 (2.3)	1	1	1	1

^∗^Significant at *P* value <0.05. ^∗∗^Significant at *P* value ≤0.001.

## Data Availability

All the data supporting the findings are within the manuscript. Additional detailed information and raw data are available from the corresponding author on reasonable request.

## Abbreviations

BMI: Body mass index
DDS: Dietary diversity score
EDHS: Ethiopian Demographic and Health Survey
FANTA: Food and nutrition technical assistant
HFIAS: Household food insecurity access scale
HHFAS: Household food access score
MDD: Major depressive disorder
OPD: Outpatient department
WHO: World Health Organization.
